# Writing Orthotic Device for the Management of Writer’s Cramp

**DOI:** 10.3389/fneur.2013.00002

**Published:** 2013-01-28

**Authors:** Narayanasarma V. Singam, Alok Dwivedi, Alberto J. Espay

**Affiliations:** ^1^Department of Neurology, UC Neuroscience Institute, Gardner Center for Parkinson’s disease and Movement Disorders, University of CincinnatiCincinnati, OH, USA; ^2^Division of Biostatistics and Epidemiology, Texas Tech University Health Sciences CenterEl Paso, TX, USA

**Keywords:** orthotic devices, medical devices, writer’s cramp, primary writing tremor, task-specific hand dystonia, writer’s cramp rating scale

## Abstract

**Background:** Oral therapies and chemodenervation procedures are often unrewarding in the treatment of focal, task-specific hand disorders such as writer’s cramp or primary writing tremor (PWT).

**Methods:** A portable writing orthotic device (WOD) was evaluated on 15 consecutively recruited writer’s cramp and PWT subjects. We measured overall impairment at baseline and after 2 weeks of at-home use with the Writer’s Cramp Rating Scale (range = 0–8, higher is worse) and writing quality and comfort with a visual analog scale (range = 0–10).

**Results:** Compared to regular pen, the WOD improved the Writer’s Cramp Rating Scale scores at first-test (*p* = 0.001) and re-test (*p* = 0.005) as well as writing quality and device comfort in writer’s cramp subjects. Benefits were sustained at 2 weeks. PWT subjects demonstrated no improvements.

**Conclusion:** WODs exploiting a muscle-substitution strategy may yield immediate benefits in patients with writer’s cramp.

## Introduction

Focal task-specific hand dystonia (writer’s cramp, WC) results from involuntary activation of antagonistic pairs of muscles of the hand, wrist, and elbow during the fine finger movements of handwriting (Ceballos-Baumann et al., 1997). WC may be generated from abnormalities in the motor circuitry within the basal ganglia and interconnected motor areas with resulting degradation of the cortical representation of the motor task and loss of normal surround inhibition for specific motor programs at the cortex (Ranawaya and Lang, 1991; Ridding, 1995; Lerner et al., 2004). Other suggested causes of WC are impaired sensorimotor integration (Serrien et al., 2000) and abnormal brainstem inhibitory mechanisms (Cohen, 1989; Chen et al., 1997). Studies have shown that WC patients may have abnormal homuncular organization of tactile sensory perception (Bara-Jimenez et al., 1998). Abnormal sensory tactile perception may elicit hyperactivation of motor areas involved in finger movement.

Writer’s cramp is challenging to treat. Anticholinergic agents (Lang, 1982), botulinum toxin (BTX) injections, and deep brain stimulation (DBS) have been used as treatment modalities for WC. Frequent problems with tolerability restrict the efficacy of anticholinergic drugs. Though studies have confirmed the clinical efficacy of BTX, its use is limited because of high cost and common adverse side effects, particularly excessive weakness limiting gross and fine-motor hand functions other than handwriting (Poungvarin, 1991; Wissel et al., 1996). The use of DBS is rarely advocated for WC as the potential risks may outweigh its restricted disability (Collins et al., 2010).

Prior evidence suggests that the application of muscle-substitution strategies may be beneficial in WC patients (Espay et al., 2005; Zeuner, 2005). Modifying the standard handwriting posture changes the handwriting technique, replacing the abnormal motor program with a normal behavior that “re-tunes” the sensorimotor circuitry. This behavior-modification strategy, also called sensory-motor retuning (SMR), has been shown to be effective in treating musician’s cramp [which shares similar pathophysiologic features with WC (Candia et al., 2005)]. Studies conducted with WC patients showed improvement after handwriting modification techniques (Ranawaya, 1991; Schenk et al., 2004). We conducted a pilot study to measure dystonic posture, writing quality, and user comfort with the use of a writing orthotic device (WOD), built with direct patient feedback over several iterations, that modifies handwriting posture in consecutively recruited subjects with WC and with another task-specific disorder, primary writing tremor (PWT). A WOD was offered to these subjects as a non-invasive, cheap to manufacture, and simple alternative intervention to chemodenervation and oral therapies.

## Materials and Methods

### Subjects

We recruited consecutively referred adult subjects with WC and PWT from the University of Cincinnati Gardner Family Center for Movement Disorders for this exploratory study. Subjects received no BTX injections or other medical interventions within 3 months prior to study enrollment. Subjects with non task-specific dystonia or tremor or who had undergone functional neurosurgical interventions, segmental, or generalized dystonia were excluded. All subjects provided Institutional Review Board-approved informed consent prior to their participation.

### Data acquisition

**Figure 1:**
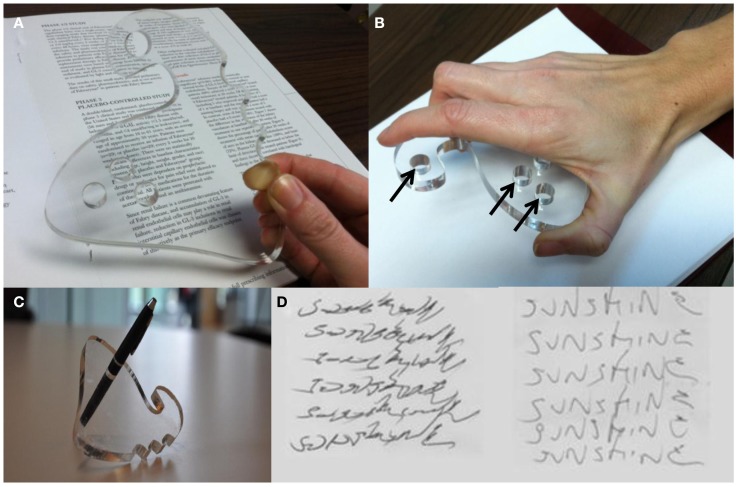
**(A)** WOD design in transparent material to allow writing visibility. **(B)** Posture of the hands during handwriting with the WOD. The pen is removable and exchangeable at any of several holes (arrows) to optimize individual handwriting techniques. **(C)** The WOD shown with a pen through one of the holes. **(D)** “Sunshine” writing task before (left) and with the use of the WOD (right) in one of the participating subjects.

Subjects were examined at baseline with a regular ballpoint pen (baseline test) followed immediately thereafter with the WOD (first-test); and reexamined 2 weeks after daily at-home use of the WOD (re-test). At each of these assessments, subjects completed standardized handwriting tasks and spiral drawing. The WOD forces a modified handwriting technique, whereby the pen is placed between the thumb and index finger using a see-through palm-sized holder (Figure 1). The standardized tasks required subjects to (1) write the word “Sunshine” six times, (2) write the sentence “Today is a sunny day” three times, and (3) to draw an Archimedes spiral. The first two tasks were designed to assess dystonic posture and the last task was used for quantification of tremor.

### Measurements

The tasks were rated using the Writer’s Cramp Rating Scale (WCRS; Wissel et al., 1996; Kruisdijk, 2006; Zeuner, 2007, 2008). The WCRS rates dystonic posturing of the elbow, wrist, and fingers; latency of dystonia and tremor; and writing speed (range = 0–30, higher is worse). Subjects also assessed the magnitude of change in writing quality and user comfort through a visual analog scale (VAS, range = 1–10; 5 = no change, ≥6 = WOD was beneficial; ≤4 = WOD was detrimental) compared to the regular pen. The subjects were asked to practice at home with the WOD to enhance comfort and ease of use prior to a reassessment (re-test) visit 2 weeks later. Subjects were also encouraged to use the WOD instead of a regular pen during this period of 2 weeks. At the re-test visit, subjects were asked to repeat the three WCRS-required tasks and estimate their VAS-based changes in writing quality and user comfort from baseline.

### The writing orthotic device

The WOD (Figure 1A) was created from 3/4″ acrylic sheets that were laser cut by a BEAM Dynamics Laser Cutter at the University of Cincinnati College of Design Art, Architecture, and Planning. It is approximately 3″ long and 3″ wide. There are three pre-drilled, 3/4″ diameter, pen-holes for placement of a pen of choice. The WOD was created after several iterations using patient feedback for ergonomics and portability using Solidworks™ CAD software. It was designed to mold to the hand between the thumb and index finger. Because of its design, it can work in either hand by turning it upside down (Figure 1B). We printed three different sizes to tailor it to a range of hand sizes. After extensive user feedback and about a dozen iterations, we chose acrylic because of its high tensile integrity and transparency, to ensure strong grip and adequate visibility of the tip of the pen and of the text being written (Figure 1C). Furthermore, the WOD fits the entire palm for increased stability and ergonomic support. When subjects write, the wrist is fixed while most of the writing movements take place proximally, at the elbow and shoulder. This forced change in the motor program was meant to provide the muscle-substitution strategy hypothesized to decrease or eliminate dystonic activity during handwriting.

### Data analysis

The mean, median, standard deviation, interquartile range (IQR), and range were used to describe quantitative variables. Frequency and proportions were used for categorical variables. The repeated scores (baseline test, first-test, and re-test) for WCRS and tremor were compared using Friedman test followed by Wilcoxon signed rank test in *post hoc* comparisons after adjusting level of significance. Comfort and writing quality scores between first-test and re-test were compared using Wilcoxon signed rank test. The statistical analyses were carried out using IBM ^®^ SPSS^®^ Statistics Base Grad Pack 19.

## Results

Fifteen subjects [9 males; 14 Caucasian; mean age, 53.7 ± 12.6 years (range, 36–76); mean age at disease onset, 33.6 ± 11.6 years (range, 15–48)] participated in the study. Two patients failed to complete study because of lack of adaptability to the WOD and two patients were lost to follow-up. Eleven subjects (64.7%) were diagnosed with WC and four (23.5%) with PWT.

There was significant improvement in WCRS scores at first-test (*p* = 0.001) and re-test (*p* = 0.005) from baseline test in WC subjects (Table 1). The magnitude of improvement was sustained, as demonstrated by similar scores between first-test and re-test at 2 weeks. Although tremor scores were reduced, the difference was not significant in PWT subjects (*p* = 0.057).

**Table 1:** **Comparisons of WCRS and tremor scores**.

Variables	Baseline (IQR)	First-test median (IQR)	Re-test median (IQR)	*p*-Value
WCRS (*n* = 11)	7 (6, 8)	3 (1, 4.3)	2 (2, 5)	<0.001
Tremor (*n* = 4)	3 (1.5, 5.5)	1 (0, 3.5)	2 (0, 3)	0.057

*IQR, interquartile range*.

Writing comfort and quality scores improved with the WOD compared with the regular pen at first-test (mean, 6.2 ± 2.2 vs. 7.7 ± 1.6, respectively). These variables did not significantly change at re-test (*p* = 0.59 vs. 0.77, respectively). A dramatic improvement in writing quality could be observed in selected subjects (Figure 1D).

## Discussion

Our pilot data suggest that the use of an orthotic handwriting device can improve handwriting in patients with WC. The benefit of WODs on WC may result from the application of a muscle-substitution strategy, by altering the hand and finger posture during handwriting, independently or through an open-loop sensory feedback mechanism. The improvement of handwriting in WC but not in PWT patients by altering the aberrant motor program further emphasizes that PWT is not a form of focal dystonia, adding to the growing body of evidence supporting the concept that PWT and WC are distinct task-specific disorders, with different pathophysiology and response to treatment (Espay and Chen, 2011).

Several studies have suggested that altering hand posture modify the sensorimotor circuits and prevent excessive activation of antagonistic muscle groups involved in WC (Zeuner, 2005; Espay and Chen, 2011). WODs that alter handwriting posture and technique have therapeutic potential in treating focal, task-specific dystonias and can be cheap and safe alternatives to chemodenervation and oral anticholinergic drugs. Abnormal patterns of supplementary motor area and primary sensorimotor cortex activation in WC have been identified using fMRI (Oga et al., 2002), which may help in future correlative studies.

To quantify the severity of symptoms, we relied on the WCRS, previously used in various WC studies (Kruisdijk, 2006; Zeuner, 2007, 2008). We found that the effects of the WOD were immediately apparent at the baseline visit. Furthermore, the magnitude of benefits was sustained at the 2 week reevaluation, without additional gains made beyond those observed at the outset. The quality and comfort scores were adequate at the initial visit with no additional improvements documented at the reevaluation, suggesting that training may not be necessary for accrual of benefits, although further improvements may be possible with a longer training period.

The WOD to alter handwriting posture was created in-house and fine-tuned over several cycles with patient-generated feedback. Additional feedback was collected from physicians, physical therapists, and biomedical engineers. One prior study assessing the effect of an orthotic device for WC was deemed “too bulky” (Ranawaya, 1991). For this reason, the WOD was designed to easily fit into anyone’s pocket for mobility. Nevertheless, exit feedbacks provided by our subjects suggested that the WOD was, at times, still awkward to use in public and this may limit its widespread use. Given more time and practice, subjects may feel less awkward. Furthermore, some subjects suggested that the device was too rigid and, at times, it was hard to see what was being written. This may explain the lower than expected writing quality and comfort scores. An improved design that makes the device more flexible and customizable with a variety of pens may improve scores. There were several limitations to this study. Because it was an exploratory study, sample population was small and the data was analyzed unblinded. Although the study design limits positive conclusions until verified with a larger sample size, it demonstrates that use of WODs may benefit patients with WC. Although logistically complicated, only double-blind randomized trials on larger samples of WC patients can ascertain the true efficacy of this and other non-invasive techniques to treat WC. Another perceived limitation with regards to this study is that the time for follow-up was of only 2 weeks. Longer training and follow-up might have further improved quality and speed of writing experienced by the subjects and provided a more accurate estimate of intolerability to the WOD.

In sum, the WOD is a cost-effective, simple, and non-invasive treatment alternative to chemodenervation and DBS for the management of the restricted disability of WC. Larger sample sizes will be necessary to confirm these preliminary findings and determine the “dose” of WOD required for optimal benefits.

## Conflict of Interest Statement

Dr. Espay has received grant support from Medtronic, CleveMed, Davis Phinney Foundation, and Michael J. Fox Foundation; personal compensation as a consultant for Solvay, TEVA, Abbott, and Chelsea Therapeutics; honoraria from Novartis, the American Academy of Neurology, and the Movement Disorders Society; and royalties from Lippincott Williams and Wilkins and Cambridge. Narayanasarma V. Singam and Alok Dwivedi declares that the research was conducted in the absence of any commercial or financial relationships that could be construed as a potential conflict of interest.
